# Improving access to medicines for non-communicable diseases in rural primary care: results from a quasi-randomized cluster trial in a district in South India

**DOI:** 10.1186/s12913-021-06800-x

**Published:** 2021-08-04

**Authors:** Manoj Kumar Pati, Upendra Bhojani, Maya Annie Elias, Prashanth N. Srinivas

**Affiliations:** 1 Karnataka Health Promotion Trust, IT park, 5th floor, No. 1-4, Rajajinagar Industrial Area, behind, KSSIDC admin. office, Rajajinagar, Bangalore, Karnataka 560044 India; 2 PhD scholar, University of Antwerp, Antwerp, Belgium; 3 Institute of Public Health, 3009 II-A Main, 17th Cross Banashankari 2nd Stage KR Road, Bangalore, Karnataka 560070 India

**Keywords:** Access to medicines, Noncommunicable diseases, Primary health care, Cluster-randomized controlled trial, Diabetes and hypertension, Out-of-pocket Health expenditure

## Abstract

**Background:**

A large proportion of non-communicable diseases (NCDs) are treatable within primary health care (PHC) settings in a cost-effective manner. However, the utilization of PHCs for NCD care is comparatively low in India. The Access-to-Medicines (ATM) study examined whether (and how) interventions aimed at health service optimization alone or combined with community platform strengthening improve access to medicines at the primary health care level within the context of a local health system.

**Method:**

A quasi-randomized cluster trial was used to assess the effectiveness of the intervention (18 months) implemented across 39 rural PHCs (clusters) of three sub-districts of Tumkur in southern India. The intervention was allocated randomly in a 1:1:1 sequence across PHCs and consisted of three arms: Arm A with a package of interventions aimed at health service delivery optimization; B for strengthening community platforms in addition to A; and the control arm. Group allocation was not blinded to providers and those who assessed outcomes. A household survey was used to understand health-seeking behaviour, access and out-of-pocket expenditure (OOP) on key anti-diabetic and anti-hypertension medicines among patients; facility surveys were used to assess the availability of medicines at PHCs. Primary outcomes of the study are the mean number of days of availability of antidiabetic and antihypertensive medicines at PHCs, the mean number of patients obtaining medicines from PHC and OOP expenses.

**Result:**

The difference-in-difference estimate shows a statistically insignificant increase of 31.5 and 11.9 in mean days for diabetes and hypertension medicines availability respectively in the study arm A PHCs beyond the increase in the control arm. We further found that there was a statistically insignificant increase of 2.2 and 3.8 percentage points in the mean proportion of patients obtaining medicines from PHC in arm A and arm B respectively, beyond the increase in the control arm.

**Conclusion:**

There were improvements in NCD medicine availability across PHCs, the number of patients accessing PHCs and reduction in OOP expenditure among patients, across the study arms as compared to the control arm; however, these differences were not statistically significant.

**Trial registration:**

Trial registration number CTRI/2015/03/005640. This trial was registered on 17/03/2015 in the Clinical Trial Registry of India (CTRI) after PHCs were enrolled in the study (retrospectively registered). The CTRI is the nodal agency of the Indian Council of Medical Research for registration of all clinical, experimental, field intervention and observation studies.

**Supplementary Information:**

The online version contains supplementary material available at 10.1186/s12913-021-06800-x.

## Background

India accounts for the largest share (66%) of deaths from non-communicable diseases (NCDs) in the South-East Asian region [1]. Even within the country, NCDs are estimated to have contributed to 5.8 million deaths (61% of all deaths) in 2017 [2]. Despite a relatively good economic growth rate (7-8% annually), demographic, social, and epidemiological transitions have contributed to an increase in NCD prevalence in both rural-urban [1, 3], rich-poor [4–6], and old and young populations [7]. Rising NCDs also translates into high healthcare visits and expenses: 40% of all hospital visits and 35% of all outpatient visits in the country [8, 9]. In terms of the economic impact of NCDs, India is estimated to have lost $237 billion (in 1998 constant international dollars) between 2006 and 2015 from premature deaths due to heart disease, stroke, and diabetes; in fact, deaths from cardiovascular disease alone in India account for the ‘highest loss in potentially productive years of life’ of all countries in the world [10]. As per the National Health Accounts of India, out-of-pocket (OOP) expenditure accounted for 64% of the total health expenditure in 2016 [11]; a high portion (55%) of these payments were on outpatient care, mostly for purchasing medications. OOP expenditure associated with NCDs is often higher than for other conditions and leads to financial catastrophe for many families who end up paying a substantial portion of their monthly income on NCD medications. A 2012 study in southern India reported that 70% of households made OOP payments for outpatient care for NCDs, and 16% suffered financial catastrophes. OOP spending doubled the number of people living below the poverty line in the study area in one month [12].

A large proportion of NCDs are treatable within primary health care settings in a cost-effective manner, and associated morbidity and mortality is preventable [13]. The principles of integration, community participation and opportunities for intersectoral collaboration offered by the primary health care approach are well suited to address such conditions [14]. Primary health centres (PHCs) are often the first point of care for the patient and therefore are strategically very important in reaching larger populations with minimal resources. The government of India launched a national NCD programme called the National Programme for Prevention and Control of Cancer, Diabetes, Cardiovascular Diseases & Stroke (NPCDCS) in 2008. The NCD programme aims to integrate health promotion, early diagnosis, treatment and referral and further facilitate partnerships with the private sector to address the rising burden of NCDs [15]. Various health system readiness issues have been posited as possible reasons for the poor availability and utilization of NCD care at PHCs [16–21]. Despite their widespread distribution and recent high-level commitments to providing NCD care at PHCs [22, 23], the utilization of PHCs for NCD care is comparatively low in India [24] due to issues related to the availability of required medicines and diagnostics [25] and trust in the quality of care [26].

The Access to Medicines (ATM) study tried to understand whether (and how) interventions aimed at health service optimization alone or combined with community platform strengthening improve access to medicines at the primary health care level within the context of local health system PHC settings in a rural district in southern Karnataka [27]. The study sought to apply a health systems lens to understand whether (and how) improvement of NCD care in PHCs could result in decreasing OOP on medicines. Using iterations of qualitative inquiry, we previously [25] explored possible contextual factors that affect the implementation and effectiveness of interventions in ATM studies. In this paper, the results of the effectiveness of ATM study interventions are presented.

## Methods

### Study objectives

This study aimed to understand whether (and how) interventions aimed at health service optimization alone or combined with community platform strengthening could improve the availability of quality generic medicines at PHCs, access to and utilization of generic medicines among patients with diabetes and hypertension and reduce out-of-pocket expenses among patients with NCDs at the primary health care level within the context of a local health system.

### Hypothesis

A package of community-level and health service-level interventions could result in improved availability of quality NCD generic medicines at PHCs, access and utilisation of such medicines among patients and reduction in out-of-pocket expenses on these medicines.

### Study setting

The study was implemented across PHCs of three *talukas* (administrative subdivisions of districts): Sira, Koratagere, and Turuvekere of Tumkur district in southern Karnataka. Tumkur is the second-largest district in the state, with an area of 10,598 km^2^ and a population of 2.67 million, of which approximately 30% were in urban areas in 2011 [28]. Tumkur is comparable to many other districts in the country in terms of a mix of government and private (ranging from single-doctor clinics to corporate chains of secondary- and tertiary-level hospitals) formal and informal healthcare providers. While health care infrastructure, especially the availability of adequate human resources, was a challenge across government PHCs, the quality and standard of care varied widely across private health care facilities. In terms of socio-economic and development indicators, Tumkur could be classified as being one of the average performance districts among the 30 districts of Karnataka state [29]. The rationale behind selecting Tumkur as the study district, health services structure in Tumkur and characteristics of the government and private health care system in this district are described in detail in the study protocol [27].

Following a rapid assessment of the performance of the local health system at the *taluka* level using the health systems dynamics framework by Olmen et al. [30], three talukas (of the 10) were excluded for not having the necessary system preparedness for the intervention. Of the remaining seven *talukas*, we randomly selected three talukas if PHCs within these talukas will all have comparable levels of readiness for implementing interventions proposed in the ATM study. The selection process is described in detail in the protocol [27]. All 39 PHCs across the three selected talukas were randomly allocated to one of three intervention arms of the study in a 1:1:1 ratio.

### Study design and tools

The ATM study was a mixed-methods study with baseline-endline quantitative surveys to assess the effectiveness and a qualitative theory-driven inquiry to explore the implementation process and contextual factors. In this paper, we present results only from the quantitative part of the study. The qualitative results have been published elsewhere [25, 26].

The quantitative survey is a before (baseline survey)-after (endline surveys) experimental design and focused mainly on identifying the determinants of improved (if any) access to medicines for diabetes and hypertension. The household survey was conducted to understand the health-seeking behaviour, access and expenditure on anti-diabetic and anti-hypertension medicines; households were picked through systematic random sampling. We conducted the surveys across PHCs as well to assess the availability of key antidiabetic and antihypertensive medicines in the previous year.

For household- and facility-level surveys, we used adapted versions of standardized World Health Organization (WHO) survey tools for household surveys and Level II facility survey tools, respectively, from the ‘WHO operational Packages for Monitoring and Assessing Country Pharmaceutical Situations’ [31]. The tools were finalised after two rounds of piloting at PHCs in an adjacent district by trained data collectors. In addition to the household and facility survey, quarterly visits to PHCs were made to collect data on the implementation of the intervention. We prepared narrative reports of each visit; key insights from these reports were compiled and analysed. We also conducted a quality test on two key antidiabetic and two antihypertensive medicines (two). Generic and branded medicines were sampled from both government and private facilities across the study talukas. While the details of such medicine sampling and medicine quality tests could be accessed from a study protocol paper [27], the results of the quality tests are published elsewhere [26].

### Household survey sampling strategy

Cluster size (number of households in the cluster) was calculated using standard equations (Additional file 1). Houses with a patient self-reporting either diabetes and/or hypertension were selected. We followed a longitudinal cohort approach. We visited the same households as in the baseline survey during the endline survey; however, we followed a sample replacement strategy during the endline survey to maintain baseline sampling probability. We replaced the households lost to follow-up with new households. The sampling strategy is described in detail in Additional file 1.

### Intervention

The intervention commenced in May 2014 and was implemented over 18 months until November 2015. The intervention PHCs were randomly allocated to one of the three intervention arms. The PHCs in arm A received a package of interventions aimed at health service delivery optimization, arm B consisted of a package of interventions aimed at strengthening community participation platforms in addition to interventions in arm A, and PHCs in arm C received no intervention other than those that are being implemented in all government PHCs.

Arm A package of interventions included training of PHC staff (doctors, pharmacists, laboratory technicians, staff nurses) on standard treatment protocols for the diagnosis and management of diabetes and hypertension, introduction of patient-retained medical records and PHC-based records for registration, follow-up of diabetes and hypertension patients, advocacy and coordination at the state, district and taluka levels to ensure a continuous supply of medicines to the PHCs. The training of PHC staff was conducted for 26 PHCs (excluding control PHCs) with a refresher in − 6- months interval to reinforce the knowledge and skills. While the training for doctors aimed at building their capacity on rational prescription, understanding the importance of prescribing generic medicines, and provision of quality care in chronic disease management, training for paramedics such as pharmacist was focused on proper indenting, storage and dispensing of generic NCD medicines. Patient-retained medical records aimed at promoting access and utilisation of generic medicines among patients besides providing health education regarding diabetes and hypertension. PHC, as well as project staff, were sensitised for regular patient follow-up on medication adherence and refill stock by visiting the PHCs once a month. Advocacy with district and state-level health authority aimed at the regular supply of generic NCD medicines to these intervention PHCs. Arm B package of interventions included development and dissemination of awareness materials, formation of patient groups, and meeting with Arogya Raksha Samiti (ARS) members on matters related to diabetes and hypertension care in addition to all interventions of Arm A. Awareness materials such as posters, brochures and pamphlets promoted access to these PHCs for free generic NCD medicines, patient groups acted as a team to discuss the importance of generic NCD medicines, addressing challenges of generic NCD medicines stock at the nearest PHCs, and above all importance regular medication adherence. ARS meetings focused on local fund use and fund flow for procuring generic NCD medicines for PHCs at times when regular supplies got hampered. Further details of the package of activities for the intervention, the justification for the design of the intervention and how they were developed are available in the study protocol [27].

### Randomization, allocation concealment and blinding

PHCs (clusters) were randomized using a simple random sampling method. PHC enrolment and assignment to intervention arms was performed based on the random numbers generated using an open-source tool (random.org^1^). The random allocation sequence was initially concealed to the researchers before the intervention was assigned to each of the three study arms. All 39 PHCs were numbered from 1 to 39. Random numbers between 1 and 39 were generated using the open-source tool. Each generated random number was kept sequentially inside the three envelopes numbered from 1 to 3. After the allocation was over, the intervention was decided for each of the three envelopes as study arms A, B or the control arm. The allocation was concealed to the researchers as well as participants (providers at PHC and patients) before the intervention was assigned. Researcher A generated the random numbers, Researcher B sequentially allocated the random numbers generated to one of the three envelopes, and at a later point in time, a third researcher (Researcher C) assigned intervention to each of the numbered envelopes. During this process, all three researchers were unaware/blinded to each other’s activity. However, after the assignment of the intervention, all researchers were not blinded during the data collection and analysis process; similarly, it was not possible to blind providers at PHC and patients to the intervention allocation. Individual patients were selected through a systematic random sampling of households across each PHC as a cluster. We obtained consent both from individual patients and PHC medical officers before the respective surveys.

### Variables

The study variables were briefly categorized into dependent variables or outcome indicators and independent sociodemographic variables. These variables are further presented in detail (definition, how measured, source of information) in Additional file 2.

#### Dependent variables

The primary outcome indicators were measured at both the facility and individual levels. Facility-level indicators include the mean number of days of availability of key generic NCD medicines at PHC, and individual-level indicators are the proportion of patients obtaining medicines from PHCs, OOP expenses among patients and the mean number of days of medicine availability at the household level. Secondary outcome indicators are the proportion of PHCs where a trained doctor was available throughout the intervention period, proportion of PHCs where a trained pharmacist was available throughout the intervention period, proportion of PHCs where a functional laboratory was there, proportion of PHCs with NCD registers, the proportion of PHCs with an active NCD patient group, the proportion of ARS meetings where NCD care and medicine availability situation at PHCs were discussed and proportion of patients aware on generic medicines. For the primary outcomes, data were collected at baseline survey and the endline survey, and for the secondary outcomes that were based on health system factors, data were collected quarterly through facility audits.

#### Independent variables

Sociodemographic variables such as age, sex, marital status, occupation, disease conditions, education and monthly income were independent variables.

### Data management and analysis

Epidata was used for data entry. Ten per cent of the data were randomly verified by the supervisor for quality. In the case of systematic errors, the remaining forms were also verified and corrected. Data were then exported from Epidata to Microsoft Excel, and final data cleaning was completed. The dataset is available (see data availability statement).

We used SPSS (Statistical Package for Social Science) for data analysis (SPSS version 20). Apart from the univariate and bivariate analyses, we analysed the intervention effect using an intention-to-treat analysis. Independent variables, such as sociodemographic characteristics, were compared to assess comparability across intervention arms using t-tests and chi-square statistics at baseline and endline survey.

The randomization is at the PHC level for the delivery of the intervention. We used the intention-to-treat analysis approach to analyse differences in outcomes across three intervention arms based on the assumption of a negligible number of crossover events. Reach to each component of the facility interventions and the community interventions was analysed separately. We also assessed the reach of the intervention in terms of health service utilization among NCD patients whom we were able to follow up from baseline to endline survey. We analysed the treatment effect through difference-in-difference analysis (DID) using STATA version 12. We adjusted for key confounders such as taluka, distant-based clusters, time to reach PHCs and sociodemographic variables in the adjusted DID regression analysis. Taluka, PHC, distant-based clusters, villages and households could be seen as multistage clusters with individuals as elementary sampling units. We analysed data further to account for clustering at the four-stage-based cluster level, such as taluka, PHC, distant-based clusters, and villages. We did not account for household-level clustering, as the patient-to-house ratio at both baseline and endline survey were close to 1. We performed clustering analysis by using survey commands in STATA (16 MP), where the dataset is defined as a four-stage clustered sample. Information on drug availability was obtained from the PHC medicine registers. Stock-out was assessed for a period of 365 days preceding the date of visit to the PHC. Mean availability days in a year for two key antidiabetic medicines (Metformin 500 mg tablet and Glibenclamide 5 mg tablet) and two antihypertensive medicines (Amlodipine 5 mg tablet and Atenolol 50 mg tablet) was compared across intervention arms. For each PHC, the maximum number of drug availability days was considered for antidiabetic and antihypertensive medicines. Mean and standard errors were estimated by linear regression as part of the difference-in-difference analysis. In addition to calculating the unadjusted difference-in-differences, we used covariates such as *taluka*, cluster, age, gender, education, occupation, home to PHC distance, and types of disease (diabetes, hypertension, both diseases) to calculate adjusted difference-in-differences in outcomes. The research team visited each PHC (including the control PHCs for routine observation) at least three times during the intervention period. Observations from these visits were instrumental in describing the role of predictors in reach and the effectiveness of the intervention.

### Ethical considerations

Ethics clearance was obtained from the WHO ethics review committee and institutional ethics committee of the Institute of Public Health, Bangalore (India). We also sought permission from the state department of health and family welfare for implementing the intervention and collecting facility-level data from PHCs. Informed written consent was sought from all participants of the surveys. Participation in the survey was voluntary, and no compensation was provided to the participants. All personally identifying information was removed from the datasets and manuscript to ensure confidentiality.

## Results

### Baseline and Endline survey

**Fig. 1:**
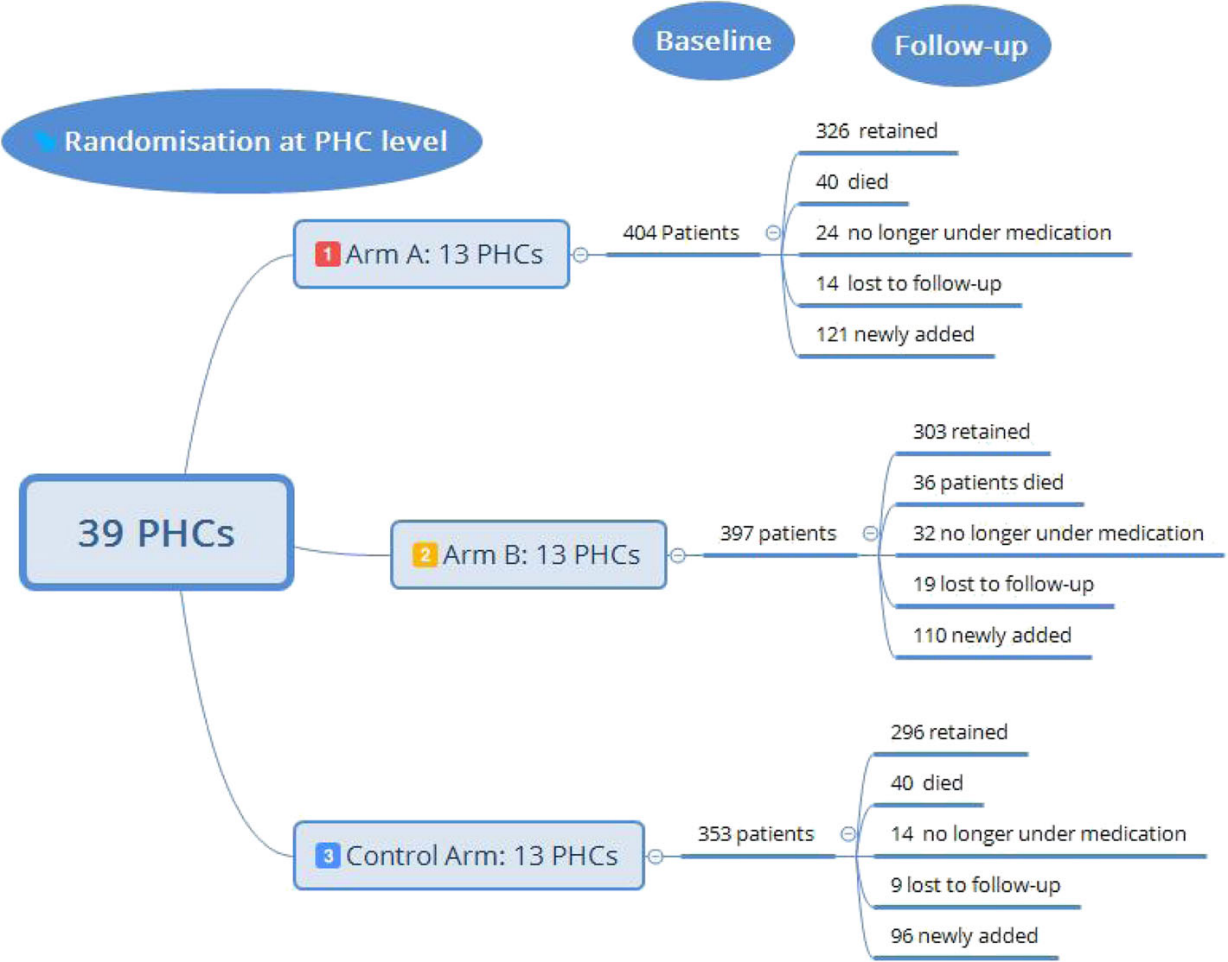
Follow-up of patients across study arms in the Access to Medicines (ATM) study; the number of patients in the baseline survey was 1154, and the number of patients in the end-line survey was 1252

The baseline survey was conducted in 1069 households across all 39 PHCs across three *talukas*: Sira, Koratagere and Turuvekere (13 PHCs each in three arms: A, B and C). We were able to resurvey 96% of patients from the baseline survey, with 4% lost to follow-up (Fig. 1); a total of 327 patients were newly recruited to the study at the end-line survey. The number of patients finally considered for analysis across three study arms in the endline survey is presented below (Fig. 1). We retained the original group allocation of PHCs and patient allocation across study arms in the final analysis; however, new patients were added to each of the three study arms.

The intervention was implemented over 18 months (May 2014 until November 2015); baseline and endline surveys were 6 months of duration each before and after the intervention period, respectively.

### Socio-demographic characteristics

The baseline and endline survey populations were comparable in terms of sociodemographic characteristics (see Table 1: Additional file 3). The households were also comparable in terms of their possession of assets (two-wheelers, fan, television sets) and amenities (tap water, electricity, etc.). Geographical access marginally improved during the 2 years from baseline to the end-line survey. Thirty-six per cent of respondents said they took more than an hour to reach PHC (43%, baseline).

### Primary study outcome

#### Improvement in the availability of quality generic medicines at PHCs

Post-intervention, the mean number of days of key medicines availability for both diabetes and hypertension increased more across the study arm A PHCs and arm B PHCs compared to the increase across control PHCs. The adjusted difference-in-difference estimates showed an increase of 31.5 and 11.9 days for diabetes and hypertension medicines respectively in the study arm A beyond the increase in the control arm from baseline to endline survey; there was an increase of 17.8 days of availability for diabetic medicines and a reduction of 1.5 days for hypertensive medicine across arm B beyond the increase in the control arm (Table 2: Additional file 4). However, these differences were not statistically significant.

#### Improvement in access and utilisation of medicines among patients with diabetes and hypertension

There was a statistically insignificant increase of 2.2 and 3.8 percentage points in the mean proportion of patients obtaining medicines from PHC for the study arm A and arm B respectively, beyond the increase in the control arm. There was an insignificant decrease of less than 1 day in the mean days of monthly medicine procurement/stock by patients in both arm A (0.57 days) and arm B (0. 75 days) as compared to control from baseline to endline survey (Table 2: Additional file 4).

### Reduction in out-of-pocket expenses among patients with NCDs

There was an insignificant decrease of 21.61 Indian rupees (~ 0.29 USD) and 65.16 rupees (~ 0.89 USD) in out-of-pocket (OOP) expenditure on NCD medicines across arm A and B respectively, beyond the decrease in the control arm (Table 2: Additional file 4).

Overall we found all these changes were not statistically significant. After adjusting for covariates, the amount and direction of change in effectiveness remained the same and were not statistically significant (Table 2).

### Role of clustering

Additional file 5 presents regression results from DID regression, accounting for four-stage clustering in STATA (16 MP). There are hardly any variations in estimates (more so for standard errors, *p* values) after adjusting for clustering. The overall results remain similar, and the effects are in the same directions.

### Key predictors of the study outcome

*Taluka* was found to be a significant predictor across all outcomes. The differences in health system performance at the taluka level could have influenced the reach and effectiveness of the intervention in different ways; other socioeconomic and taluka-specific factors could also explain this. Patients in Sira and Koratagere taluka had better availability than patients in Turuvekere. Similarly, nearly 60% of patients in Sira and Koratagere visited their PHCs, while it was only 40% for Turuvekere. The mean number of days of medicine availability with the patients did not differ at all from baseline to –endline survey across study arms. Irrespective of the intervention and intervention period, the median days of availability of medicines at home stayed at 20 days.

We found age to be a significant predictor for the mean number of days of availability of key NCD medicines at home. Older patients tend to procure NCD medicines for longer days. Occupation (those who were employed tended to spend more and procure medicine for more days) was significantly associated with OOP on NCD medicines and the mean number of days for medicine availability at home. We found education to be a significant predictor for OOP and the source from which medicines were procured. With an increase in education, higher reliance on private facilities for medicines and incurring more OOP was evident. The type of disease and medicine source were significantly associated with OOP. Patients with diabetes spent more than patients with hypertension, and patients with two or more NCDs spent more than patients with a single disease. Patients spent more at private facilities, as expected. Time taken to reach PHCs was significantly associated with the number of days of availability of medicines with NCD patients. Those taking more time to reach a particular facility tended to procure more days of medicines.

### Secondary outcomes

**Fig. 2:**
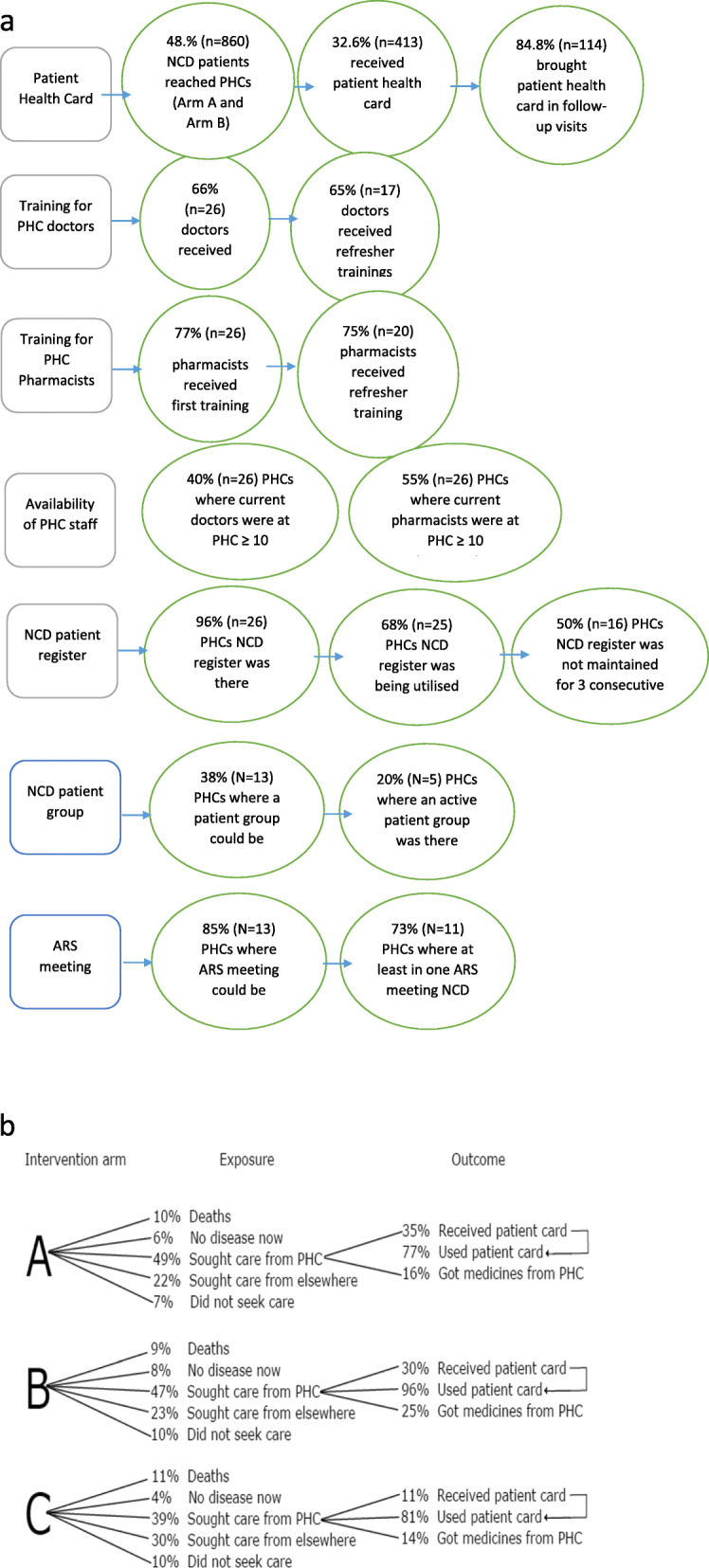
a. Reach of the intervention b. Health care utilization across the two intervention arms

Secondary outcomes are based on the intervention elements and are majorly focused on the reach of the intervention and the overall utilisation of NCD care at PHCs. Reach to the intervention was below par, especially for the community-level package of interventions (Fig. 2a). Overall, 413 (48%) out of 860 patients in study arm A and B accessed these PHCs during the intervention period; 49% of patients sought care from PHCs in study arm A compared to 47% in arm B. Only one hundred and fourteen (13.2%) patients in these intervention arms could obtain the patient retained records/cards; however, 97 patients (84.8%) among them brought those records during their follow-up visits. While in 17 (66%, *n* = 26) PHCs, doctors received the training, 11 (65%) among them could receive the refresher training. Similarly, in 20 (77%, *n =* 26) PHCs, pharmacists received the training and 15 (75%) among them could attend the refresher training. The follow-up profile of patients who reached intervention PHCs is presented in Fig. 2b.

### Role of health system factors

The total number of routine visits made to all PHCs during intervention was 106. During these visits, an audit of relevant documents (stock record, registers, records) and observation of processes (prescription practices, medicine storage and dispensing) were conducted. We found that few PHCs (six out of 39 total) performed better than the others. These PHCs mainly had stable, motivated staff with a keen interest in providing NCD care; there were larger health system challenges that constrained effective implementation of the intervention (Additional file 6).

## Discussion

This study aimed to understand whether (and how) interventions aimed at health service optimization alone or combined with community platform strengthening improve access to medicines at the primary health care level within the context of a local health system. The interventions did not achieve the desired significance level of differences across study arms. Overall, we found that Arm-B, which received both types of interventions, fared marginally better than the other two study arms in terms of key intervention outcomes such as medicine availability at home and PHCs; however, the difference was not statistically significant.

The intervention effects were not uniform within the study arms beside it being ineffective. Another research article reporting qualitative research findings from this study highlights key barriers and contextual factors associated with non-uniform effects and ineffective intervention [25]. Some PHCs were able to overcome health system barriers better than others due to the strong motivation and leadership of their staff and due to good rapport with local community participation platforms. In some cases, even with trained and motivated staff, contextual issues such as episodic medicine supplies, critical staff shortage, gaps in essential diagnostics to provide NCD care and frequent staff turnover affected the implementation of the intervention. The PHCs being part of a larger complex health system were influenced by challenges that were beyond the control of study interventions to address [25].

In addition, substantial gaps in governance at the district and state levels influenced better access to medicines to patients [25]. On the one hand, decision space available at the district level to influence better availability of medicines at the PHC level was limited; on the other hand, the state-level drug supply agency was not adequately responsive to the needs of PHCs. Even in terms of prioritizing NCDs within the primary health context, the NPCDCS programme fails to provide substantial resources to the PHC/sub-centre to make a difference in NCD care at this level [25]. The role of PHC is limited to screening, case identification and referral to higher centres. This role is also a likely influence on the lack of priority given by the PHC staff to NCD medicines while indenting and the provision of care at the health centres.

An important societal/health system factor that influences public service utilization is trust in the PHC/public system itself. In this study, we have previously reported [26] the influence of trust in medicines and public services as possible drivers of private-sector dependence for NCD care among rural populations in Tumkur. Perceptions of both healthcare providers and patients about medicines at PHCs exerted an influence on the utilization of PHCs. A lack of trust in the quality of *government medicines* that was prevalent among health workers (both public and private) and patients could influence the reach and effectiveness of health services interventions. Despite objective tests revealing the comparable quality of medicines between government and private sector, subjective perceptions of medicine quality deteriorated from baseline to endline survey. This could also explain the poor preference for PHC medicines by NCD patients despite improvements in their availability; it also explains possible reliance on outside prescriptions of medicines by PHC health workers.

The paying capacity of the study population improved during the 2 years, as evident by the decrease in the proportion of patients borrowing money or selling assets to purchase medicines and increased household income levels. This finding could also be associated with an increase in the proportion of patients obtaining medicines from PHCs. However, since the number of participants receiving free medicines from PHCs remained relatively low and similar across study arms, it could more likely mean that increased affordability may be due to an overall increase in median household income, which nearly doubled across all three arms. Increased income has often been used to finance healthcare expenditure [32].

One of the key components of both intervention arms was capacity building of the PHC staff in the management of NCDs at the primary health care level. The uptake of such a capacity-building exercise and its sustainability is influenced by several factors. Bates et al. [33] found that it took an average of 66 months for a capacity-building project to become sustainable in terms of improved knowledge and effectiveness. Since the follow-up survey in our study was conducted just a year and a half after the capacity building exercise, it is probably too early to assess the results of the intervention. In addition, the connection between capacity building and performance is not straightforward; earlier studies in the district concerning capacity building have shown the complex relationship between capacity building and various organizational and individual factors [34]. Bates et al. [33] also identified some of the challenges that influence performance following capacity building, such as high staff turnover, issues with the adoption of new activities into existing systems, and influencing policy development. These were very similar to what we found in our study. In only 40% of the PHCs, the doctor and 55% of PHCs, the pharmacists, at the follow-up survey, were available for ten consecutive months in the same PHC. This is further reflected in the fact that only around 50% of the doctors and pharmacists received both initial and refresher training.

## Conclusion

Our study reinforces the fact that access to medicines in PHCs is embedded within a complex adaptive health system, and its barriers are therefore equally complex and influenced by factors at multiple levels of the system [35]. Health service-level interventions had limited effects on improving access to medicines for patients with diabetes and hypertension, possibly due to wider societal and health system factors such as trust in public services and difficulties in ensuring the stability and capacity of human resources within public services. Although findings from this study are limited to the study geography, inferences and insights could apply to similar settings.

### Limitations

We used a quasi-randomized cluster trial design, while health facilities were randomized to one of the three study arms, the unit of analysis was at both the health facility and patient levels. However, we failed to hold on to important assumptions of RCTs beyond treatment allocation to the study arms. The following assumptions are violated, which affect the internal validation of the study:

1. **Blinding**: Blinding to treatment/intervention was not possible in this health service research due to ethical and contextual reasons. Blinding treatment allocation was known to the research team, who collected and analysed the data. However, we tried to minimize bias by treating control arm facilities and participants as equally as possible. We ensured that the frequency of follow-up visits and basic standards of care were similar for control arm facilities. While we minimized selection bias through random allocation, we tried to minimize the performance bias (differential treatment of groups) and ascertainment bias (biased assessment of outcomes), which could have resulted from failing to blind data collectors and the person doing analysis, by ensuring key study outcomes were as objective as possible and by ensuring duplicate assessment in a small sample of data by two analysts and reporting the level of agreement.

2. **Diffusion of intervention across study arm**: Providers at health facilities as well as patients who interacted across study arms and patients accessed multiple health facilities (including those in the control arm). Some control arm health facilities initiated similar interventions on their own. Working within health services implies an acceptance of lack of control over diffusion of the intervention in part or full to control arms. Indeed, our overall advocacy to improve medicine supply for NCDs at the district and state level covered all PHCs, as it would have been neither feasible nor ethical to restrict such advocacy to the intervention PHCs only.

3. **Instrumentation and testing**: It is possible that discussing with staff on key study outcomes (such as availability) at each visit caused them to react in a certain way that is different than they would have otherwise. Repeated probing/measurement of outcome also influences outcomes.

4. **Confounding**: Government medicine supplies to these health facilities were episodic; sometimes there were periods of stock out while there were periods of ample stock/good supplies.

5. **Wider systemic issues**: Frequent drug stock-outs, frequent turnover among healthcare staff, and challenges of the continuous capacity building remained perennial challenges hindering our intervention to work to its full potential.

However, we feel, though we could not follow rigorous research methods to boost the internal validity, the study had fair external validity as it showcased implementation that was close to how participants behave in real-world settings. Although we conducted an *intention-to-treat* analysis in complex health services settings such as ours, it is not possible to carefully separate the effects of individual intervention components on key study outcomes.

## Supplementary Information

**Additional file 1.** Sampling Strategy. Description of data: Description of sampling strategy employed in the Access to Medicines study

**Additional file 2.** List of study variables. Description of data: List of key study variables, how these measured and source of information

**Additional file 3:.** Table 1: Sociodemographic characteristics of people with diabetes and hypertension across study arms. Description of data: Comparison of sociodemographic characteristics of people with diabetes and hypertension between study arms

**Additional file 4:.** Table 2: Effectiveness of health service optimization and community platform strengthening on study outcomes. Description of data: Results of a difference-in-differences analysis across study arms for key outcome indicators in the baseline and endline survey

**Additional file 5.** 4-stage cluster adjusted DID results. Description of data: Results of the difference-in-differences analysis after adjusting for clustering at taluka and distant-based cluster levels

**Additional file 6.** Health System Challenges affecting Access to Medicines in the ATM Study. Description of data: List of health system-level challenges that affected the intervention implementation in the ATM study

## Data Availability

The datasets generated and/or analysed during the current study are available in the Figshare repository in an anonymised form: https://figshare.com/articles/dataset/Dataset_of_the_ATM_study_implemented_in_southern_Karnataka_primary_health_centres_to_improve_access_to_medicines_to_non-communicable_diseases/12957185

## Abbreviations

ARS: Arogya Raksha Samiti
NPCDCS: National Programme for Prevention and Control of Cancer, Diabetes, Cardiovascular Diseases & Stroke
NCDs: Non-communicable diseases
OOP: Out-of-pocket (OOP) health expenditure
PHCs: Primary health centres
SPSS: Statistical Package for Social Science
ATM: The Access to Medicines study
WHO: World Health Organization

## Footnotes

1 Available at: https://www.random.org
