# Supplementary material for: Improving access to medicines for non-communicable diseases in rural primary care: results from a quasi-randomized cluster trial in a district in South India
Source: BMC Health Serv Res. 2021 Aug 4;21:770. doi: 10.1186/s12913-021-06800-x (PMC8336076; doi:10.1186/s12913-021-06800-x)
Supplement: Supplementary file 1 — Additional file 1. Sampling Strategy. Description of data: Description of sampling strategy employed in the Access to Medicines study [file 12913_2021_6800_MOESM1_ESM.docx]

**Additional File 1**

**Sampling Strategy in the Access to Medicines Study**

The WHO household survey tool does not have NCD specific inclusion criteria. Sample size calculation was based on obtaining at least 30 households per PHC spread across three clusters (a, b and c with 10 households per cluster; see figure below). A typical PHC in Tumkur district has at least five villages in each cluster, and hence at least 1000 households in each cluster (approximately 250 households in a village).

Fig 1: Study setting of the ATM study. Map by authors. Base map derived from global administrative areas database (gadm.org) which is licensed for academic use with attribution.

**Baseline sampling strategy**

The three Talukas selected after the rapid health assessment had a total of 39 PHCs. For household survey, we randomly distributed all 39 PHCs into one of the three study arms. A cluster of households under each PHC was randomly selected based on their distance from the PHC. Villages within five kilometres distance of the PHC formed cluster a, Villages at five to ten kilometres distance formed cluster b and those villages located more than 10 kilometres away formed cluster c (Fig 1). One village per cluster for the survey which constituted up to 10 households in each village ( or within each cluster) was chosen. Households were purposively selected as having at least a patient suffering from either diabetes or hypertension. Where we did not get 10 such households in a given village we selected households from neighbouring village in the same cluster. We collected data from 1069 households across 107 clusters of 39 PHCs.

Facility survey was conducted in all the 39 primary health centres and 30 private pharmacies nearby these PHCs and 30 patient exit interviews were conducted per PHC.

**End line Household Sampling Strategy**

For this study we adopted a longitudinal cohort design. For baseline households that we lost to follow up/trace^[[1]](#footnote-1)^ we replaced new households (having a diabetes or hypertension patient) in the end line survey. In the end line survey, we also encountered death of the NCD patients in some of the baseline households. They were separately analysed and included in our sample size.

As the primary aim of the study was to compare the outcomes before and after the intervention, we followed a sample replacement strategy. The reasons behind replacement were manifold. The main reason was to maintain equal level of randomization in selecting households at baseline and end line survey as the intervention was randomized at PHC level not at the household. By replacing households, the probability of randomly selecting a household with diabetes and hypertension patient (from the same population) remained unchanged. The second reason behind replacement was to have a comparable sample in order to achieve reduction in cluster and intra-cluster (between the intervention arms) variability in sample size. The third reason of having a comparable sample was to reduce the effect of clustering of outcome in a particular cluster.

In individually randomized trials, the number of study participants randomized are large enough to ensure close comparability of intervention groups. However, in cluster randomized trials the number of clusters randomized are often small, and so randomization can’t be relied fully to achieve comparability. (Hayes & Bennett, 1999) A common strategy in such trials is to arrange the available clusters into matched pairs. Randomisation to treatment groups is then carried out within pairs, and a matched analysis can be conducted to verify for clustering and between cluster variability.

For the ATM study, unfortunately average cluster size and a proportion of primary outcome could not be estimated precisely at the start of cluster randomization but at end line using findings from baseline an estimation of appropriate sample size was determined.

Going by above logic we had modified our sample size for the end line household survey as follows.

Sample size “n” in each group of clusters is equals to,

**n= "(Zα/2+Zβ)2 [π0(1-π0) +π1(1-π1)] " /"(π0-π1)2 "**

Where π0= Proportion of a primary outcome of the study without (before) the intervention.

For example, proportion of households obtained NCD medicines from PHC at baseline survey (before the intervention) =15%=0.15

Where π1= Proportion of a primary outcome of the study with (after) the intervention.

Assuming a 15% increase from baseline, proportion of households obtained NCD medicines from PHC with (after) the intervention=30%=0.30

Where "Zα/2” and "Zβ" are the standard normal distribution values corresponding to upper tail probabilities of α/2(significance level for a two tailed test) and β (type two error-failing to detect that an effect is present) respectively. This choice of sample size provides a power of 100(1-β) % of obtaining a significant (p<α for a two sided test) difference (15% in this case) assuming that true proportion in the presence and absence of intervention are π1 and π0 respectively.

We have total nine group of matched clusters of households (three a cluster, three b cluster and three c cluster for each of the intervention arm A, B and C). Clusters are matched basing upon their common attributes that is their distance from the PHC. Cluster a households are within five kilometres of the respective primary health centre, cluster b households are in five to 10 kilometres distance and cluster c households are beyond 10 kilometres distance.

Before calculating the sample size let us clarify the expression,

**"[π0(1-π0) +π1(1-π1)] = (2/9) x S" 2x D**

Where, D= Design effect or variance inflation factor

D= 1+[m(1+cv2)-1] Þ= 1+ [10(1+0.322)-1]0.15= 2.5 accounting for clustering and variable cluster size.

S=Sample error for the outcome=0.4

m-= Actual mean cluster size at baseline=1069/107=9.9 households

cv=coefficient of variation in cluster size=0.32, Þ=Intra cluster correlation coefficient=0.15

Then, n= ("(1.96+0.84)2(0.15*0.85+0.30*0.70") /"(0.15-0.30)2" = 118.4 households for each group of cluster.

For nine such group of clusters (3 ‘a’, 3 ‘b’ and 3 ‘c’) = 118*9= 1062 households.

Since we had gone for a panel survey, we assumed a maximum of 20% households would be lost to follow up from baseline. Hence plan was to conduct the survey among 1274(1062+0.2*1062) households.

As we had exactly a total of 107 clusters in our study area, we surveyed around 12 households per cluster. (1274/107=12)

1. Households that could not be located, patient died, household locked during visit, no persons in the household were able to answer or household refused to respond. [↑](#footnote-ref-1)
