# Supplementary material for: Improving access to medicines for non-communicable diseases in rural primary care: results from a quasi-randomized cluster trial in a district in South India
Source: BMC Health Serv Res. 2021 Aug 4;21:770. doi: 10.1186/s12913-021-06800-x (PMC8336076; doi:10.1186/s12913-021-06800-x)
Supplement: Supplementary file 2 — Additional file 2. List of study variables. Description of data: List of key study variables, how these measured and source of information [file 12913_2021_6800_MOESM2_ESM.pdf]

Primary and secondary outcome of the study with analysis plan

**Primary outcome of interest**			
**Factors to be measured**	**Indicator**	**Analysis plan**	**Source of data**
Improved availability of generic anti-diabetic and anti-hypertensive drugs at PHCs	**Mean (SE) days of availability of key anti-diabetic and key anti-hypertensive medicines at PHCs** calculated from Maximum number of days of availability of either of two key anti-diabetic or anti-hypertensive medicines in a year (365 days) prior to date of survey	Intervention effect- Baseline-endline difference (Diff-in-Diff Analysis)	Facility survey data
Increased access to PHCs for Medicines patients with diabetes and hypertension	**Mean (SE) proportion of patients could obtain NCD medicines from PHCs**	Diff-in-Diff Analysis	Patient level data
Improved availability of medicines patients with diabetes and hypertension	**Mean(SE) number of days for which medicine obtained by patients**<br><br>**(Normally patients obtain medicines for 30 days from PHCs)**	Diff-in-Diff Analysis	Patient level data
Out-of-pocket expenses among patients with diabetes and hypertension	**Mean (SE) OOP in INR on NCD medicines per month**	Diff-in-Diff Analysis<br><br>Difference in median OOP among patients between intervention arms at endline.	Patient level data

**Secondary outcome of interest**			
**Factors**	**Indicators**	**Analysis plan**	**Source of data**
Better coordination among health staffs with regards to NCD patient group formation	- Percentage increase in number of patient groups formed within first quarter and last quarter of	Across intervention arms<br><br>Difference calculation	Intervention follow up data

	intervention (Only in intervention B PHCs)<br><br>- Percentage increase in number of new diabetes patients registered in last three months (from the date of visit) for a given PHC.<br><br>- Percentage increase in number of new hypertension patients registered in last three months for a given PHC .		
Better availability of staffs	-Average number of months medical officers available in last 18 months<br><br>- Average number of months pharmacists available in last 18 months<br><br>- Average number of months lab technicians available in last 18 months	Across intervention arm difference calculation	Intervention follow up data
Conduction of NCD clinics	Percentage PHCs conduct NCD camps in last three months	Across intervention arm difference calculation	Intervention follow up data
Availability of functional laboratory	Percentage PHCs having a functional laboratory	Across intervention arm difference calculation	Intervention follow up data
Record maintenance	Percentage of PHC where NCD patient 'register maintained at least for 12 months in last 18 months	Across intervention arm difference calculation	Intervention follow up data
Better dispensing by the pharmacists at PHC	Percentage NCD medicines dispensed as prescribed	Difference of aggregated percentage NCD medicines dispensed as prescribed across intervention arms	Exit interview
