# Supplementary material for: Improving access to medicines for non-communicable diseases in rural primary care: results from a quasi-randomized cluster trial in a district in South India
Source: BMC Health Serv Res. 2021 Aug 4;21:770. doi: 10.1186/s12913-021-06800-x (PMC8336076; doi:10.1186/s12913-021-06800-x)
Supplement: Supplementary file 4 — Additional file 4:. Table 2: Effectiveness of health service optimization and community platform strengthening on study outcomes. Description of data: Results of a difference-in-differences analysis across study arms for key outcome indicators in the baseline and endline survey [file 12913_2021_6800_MOESM4_ESM.docx]

**Table 2: Effectiveness of health service optimisation and community platform strengthening on study outcomes**

|  |  |  |  |  |  |  |  |  |  |  |  |  |  |  |  |  |  |
| --- | --- | --- | --- | --- | --- | --- | --- | --- | --- | --- | --- | --- | --- | --- | --- | --- | --- |
|  | **A (Health service optimisation)** | **B )(Health service optimisation +community platform strengthening)** | **C (control)** | **A-C** | **Unadjusted differences-in differences (SE)** | **Adjusted differences-in differences (SE)** | **B-C** | **Unadjusted differences-in differences (SE)** | **Adjusted^##^ differences-in differences (SE)** |  |  |  |  |  |  |  |  |
|  |  |  |  |  |  |  |  |  |  |  |  |  |  |  |  |  |  |
|  | **Mean (SE) days of availability of key anti-diabetic medicines at PHCs** | | | | | | | | |  |  |  |  |  |  |  |  |
| Baseline | 309.6 (20.1) | 323.4 (9.9) | 335.2(6.8) | -25.6 (21.7) |  |  | -11.6 (11.0) |  |  |  |  |  |  |  |  |  |  |
| Endline | 360 (3.1) | 360.4 (3.1) | 354.2 (7.1) | 5.8 (7.5) | 31.5(22.5) | 31.5(19.8) | 6.2(7.8) | 17.8 (14.3) | 17.8(12.9) |  |  |  |  |  |  |  |  |
| Difference | 50.4 | 37 | 19 | P-value^#^ | 0.16 | 0.11 | P-value | 0.22 | 0.17 |  |  |  |  |  |  |  |  |
|  | **Mean (SE) days of availability of key anti-hypertensive medicines at PHCs** | | | | | | | | |  |  |  |  |  |  |  |  |
| Baseline | 342.7 (11.8) | 325.7 (19.9) | 353.4 (6.4) | -10.7 (14.9) |  |  | -27.7 (20.5) |  |  |  |  |  |  |  |  |  |  |
| Endline | 361.5 (2.5) | 331.1 (27.8) | 360.3 (3.1) | 1.2 (4.3) | 11.9 (14.0) | 11.9 (14.1) | -29.2 (28.5) | -1.5 (34.9) | -1.5 (37.4) |  |  |  |  |  |  |  |  |
| Difference | 18.8 | 5.4 | 6.9 | P-value | 0.4 | 0.39 | P-value | 0.96 | 0.96 |  |  |  |  |  |  |  |  |
|  | **A (N=723)** | **B (N=688)** | **C (N=608)** | **A-C** | **Unadjusted differences-in differences (SE)** | **Adjusted differences-in differences (SE)** | **B-C** | **Unadjusted differences-in differences (SE)** | **Adjusted^##^ differences-in differences (SE)** |  |  |  |  |  |  |  |  |
|  |  | | | | | | | | |  |  |  |  |  |  |  |  |
|  | **Mean (SE) proportion of patients could obtain NCD medicines from PHCs** | | | | | | | | |  |  |  |  |  |  |  |  |
| Baseline | 8.2 (1.6) | 14.9 (1.8) | 9.3(1.7) | -1.1 (2.3) |  |  | 5.6 (2.7) |  |  |  |  |  |  |  |  |  |  |
| Endline | 11.4(1.6) | 18.5(1.8) | 10.8 (1.7) | 0.6 (2.3) | 1.7 (3.3) | 2.2 (3.2) | 7.7 (2.7) | 2.1 (3.8) | 3.8 (5.1) |  |  |  |  |  |  |  |  |
| Difference | 3.2 | 3.6 | 1.5 | P-value | 0.61 | 0.50 | P-value | 0.56 | 0.65 |  |  |  |  |  |  |  |  |
|  | **Mean (SE) OOP in INR on NCD medicines per month** | | | | | | | | |  |  |  |  |  |  |  |  |
| Baseline | 221.80 (30.03) | 175.994(26.14) | 224.47 (31.93) | -2.665 (43.83) |  |  | -48.47(37.81) |  |  |  |  |  |  |  |  |  |  |
| Endline | 290.14 (29.45) | 203.57(26.06) | 319.08 (32.78) | -28.94 (44.07) | -26.28 (62.16) | -21.61(60.66) | -115.51(38.29) | -67.03(53.02) | -65.16(52.63) |  |  |  |  |  |  |  |  |
| Difference | 68.34 | 27.58 | 94.61 | P-value | 0.67 | 0.71 | P-value | 0.21 | 0.23 |  |  |  |  |  |  |  |  |
|  | **Mean(SE) number of days for which medicine obtained by patients** | | | | | | | | |  |  |  |  |  |  |  |  |
| Baseline | 19.226 (0.56) | 19.75 (0.53) | 18.891 (0.59) | 0.335 (0.81) |  |  | 0.86 (0.77) |  |  |  |  |  |  |  |  |  |  |
| Endline | 21.228 (0.54) | 21.601 (0.53) | 21.600(0.61) | -0.372(0.82) | -0.707 (1.16) | -0.578 (1.14) | 0.001 (0.785) | -0.859 (1.1) | -0.751(1.095) |  |  |  |  |  |  |  |  |
| Difference | 2.002 | 1.85 | 2.709 | P-value | 0.54 | 0.61 | P- Value | 0.49 | 0.49 |  |  |  |  |  |  |  |  |

## *Means and robust standard errors are estimated by linear regression* # P-values *<0.05 **<0.01 ***<0.001
