# Supplementary material for: Improving access to medicines for non-communicable diseases in rural primary care: results from a quasi-randomized cluster trial in a district in South India
Source: BMC Health Serv Res. 2021 Aug 4;21:770. doi: 10.1186/s12913-021-06800-x (PMC8336076; doi:10.1186/s12913-021-06800-x)
Supplement: Supplementary file 5 — Additional file 5. 4-stage cluster adjusted DID results. Description of data: Results of the difference-in-differences analysis after adjusting for clustering at taluka and distant-based cluster levels [file 12913_2021_6800_MOESM5_ESM.pdf]

4-stage cluster adjusted DID results

Community Platform Strengthening Vs. Control Arm (after adjusting for clustering at taluka, PHC, distant based cluster, village level)

```
. diff medsource, t(treated) p(period)

DIFFERENCE-IN-DIFFERENCES ESTIMATION RESULTS
Number of observations in the DIFF-IN-DIFF: 1307
            Before            After
   Control: 313               305         618
   Treated: 342               347         689
            655               652
--------------------------------------------------------
 Outcome var.    | medso~e  | S. Err. |   |t|   |  P>|t|
-----------------+----------+---------+---------+--------
Before           |          |         |         |
   Control       | 0.093    |         |         |
   Treated       | 0.146    |         |         |
   Diff (T-C)    | 0.054    | 0.027   | 2.00    | 0.045**
After            |          |         |         |
   Control       | 0.111    |         |         |
   Treated       | 0.187    |         |         |
   Diff (T-C)    | 0.076    | 0.027   | 2.83    | 0.005***
                 |          |         |         |
Diff-in-Diff     | 0.022    | 0.038   | 0.59    | 0.556
--------------------------------------------------------
R-square:    0.01
* Means and Standard Errors are estimated by linear regression
**Inference: *** p<0.01; ** p<0.05; * p<0.1
```

```
. diff medsupply, t(treated) p(period)

DIFFERENCE-IN-DIFFERENCES ESTIMATION RESULTS
Number of observations in the DIFF-IN-DIFF: 1304
            Before            After
   Control: 313               303         616
   Treated: 342               346         688
            655               649
--------------------------------------------------------
 Outcome var.    | medsu~y  | S. Err. |   |t|   |  P>|t|
-----------------+----------+---------+---------+--------
Before           |          |         |         |
   Control       | 18.891   |         |         |
   Treated       | 19.751   |         |         |
   Diff (T-C)    | 0.860    | 0.774   | 1.11    | 0.267
After            |          |         |         |
   Control       | 21.525   |         |         |
   Treated       | 21.633   |         |         |
   Diff (T-C)    | 0.108    | 0.778   | 0.14    | 0.889
                 |          |         |         |
Diff-in-Diff     | -0.752   | 1.098   | 0.68    | 0.493
--------------------------------------------------------
R-square:    0.01
* Means and Standard Errors are estimated by linear regression
**Inference: *** p<0.01; ** p<0.05; * p<0.1
```

```
. diff ncdmedcost, t(treated) p(period)

DIFFERENCE-IN-DIFFERENCES ESTIMATION RESULTS
Number of observations in the DIFF-IN-DIFF: 1301
            Before            After
   Control: 313               304         617
   Treated: 342               342         684
            655               646
--------------------------------------------------------
 Outcome var.    | ncdme~t  | S. Err. |   |t|   |  P>|t|
-----------------+----------+---------+---------+--------
Before           |          |         |         |
   Control       | 224.473  |         |         |
   Treated       | 175.994  |         |         |
   Diff (T-C)    | -48.479  | 37.743  | -1.28   | 0.199
After            |          |         |         |
   Control       | 322.036  |         |         |
   Treated       | 202.655  |         |         |
   Diff (T-C)    | -119.381 | 38.033  | 3.14    | 0.002***
                 |          |         |         |
Diff-in-Diff     | -70.903  | 53.582  | 1.32    | 0.186
--------------------------------------------------------
R-square:    0.01
* Means and Standard Errors are estimated by linear regression
**Inference: *** p<0.01; ** p<0.05; * p<0.1
```

Health Systems Optimization vs. Control Arm

```
. diff medsource, t(treated) p(period)

DIFFERENCE-IN-DIFFERENCES ESTIMATION RESULTS
Number of observations in the DIFF-IN-DIFF: 1346
            Before         After
   Control: 313            305          618
   Treated: 354            374          728
            667            679
```

Outcome var.	medso~e	S. Err.	|t|	P>|t|
Before				
Control	0.093			
Treated	0.082			
Diff (T-C)	-0.011	0.023	-0.46	0.645
After				
Control	0.111			
Treated	0.115			
Diff (T-C)	0.003	0.023	0.15	0.880
Diff-in-Diff	0.014	0.033	0.43	0.665

```
R-square:    0.00
* Means and Standard Errors are estimated by linear regression
**Inference: *** p<0.01; ** p<0.05; * p<0.1

. diff medsupply, t(treated) p(period)

DIFFERENCE-IN-DIFFERENCES ESTIMATION RESULTS
Number of observations in the DIFF-IN-DIFF: 1343
            Before         After
   Control: 313            303          616
   Treated: 354            373          727
            667            676
```

Outcome var.	medsu~y	S. Err.	|t|	P>|t|
Before				
Control	18.891			
Treated	19.226			
Diff (T-C)	0.335	0.811	0.41	0.680
After				
Control	21.525			
Treated	21.134			
Diff (T-C)	-0.391	0.809	0.48	0.629
Diff-in-Diff	-0.725	1.145	0.63	0.527

```
R-square:    0.01
* Means and Standard Errors are estimated by linear regression
**Inference: *** p<0.01; ** p<0.05; * p<0.1

. diff ncdmedcost, t(treated) p(period)
```

```
. diff ncdmedcost, t(treated) p(period)
```

DIFFERENCE-IN-DIFFERENCES ESTIMATION RESULTS

Number of observations in the DIFF-IN-DIFF: 1342

	Before	After	
Control:	313	304	617
Treated:	354	371	725
	667	675	

Outcome var.	ncdme~t	S. Err.	|t|	P>|t|
Before				
Control	224.473			
Treated	221.808			
Diff (T-C)	-2.665	43.749	-0.06	0.951
After				
Control	322.036			
Treated	291.620			
Diff (T-C)	-30.416	43.622	0.70	0.486
Diff-in-Diff	-27.751	61.781	0.45	0.653

```
R-square:    0.01
* Means and Standard Errors are estimated by linear regression
**Inference: *** p<0.01; ** p<0.05; * p<0.1

.
```
