# Supplementary material for: Improving access to medicines for non-communicable diseases in rural primary care: results from a quasi-randomized cluster trial in a district in South India
Source: BMC Health Serv Res. 2021 Aug 4;21:770. doi: 10.1186/s12913-021-06800-x (PMC8336076; doi:10.1186/s12913-021-06800-x)
Supplement: Supplementary file 6 — Additional file 6. Health System Challenges affecting Access to Medicines in the ATM Study. Description of data: List of health system-level challenges that affected the intervention implementation in the ATM study [file 12913_2021_6800_MOESM6_ESM.pdf]

Health System Challenges affecting Access to Medicines in the ATM Study

1. Episodic availability of NCD medicines and logistics

Although the overall stock-out of medicines decreased from baseline to endline, medicine stock, particularly those for diabetes and hypertension, remained episodic across PHCs of all study arms during a greater part of the intervention period. This issue occurred despite the availability of stocks at the district level most of the time. In many PHCs, this appeared to be due to higher priority given to antibiotics during medicines indent process. The availability of laboratory supplies such as glucometer strips was also poor in most PHC laboratories.

2. Outside prescription of medicines at PHCs

From our household survey, we found that although nearly half of the NCD patients visited PHCs during our intervention period, only 11% of them had obtained medicine from these PHCs anytime ever during this period. Most patients purchased medicines from private pharmacies near PHCs through prescriptions provided by PHC doctors.

3. Acceptability of awareness material and patient record books

Awareness materials on common NCDs, including posters on lifestyle modification, was appreciated widely by health workers and were well utilized; however, those on promoting generic medicines received a mixed response. PHC doctors reported positive feedback on the acceptability of patient-retained health cards for diabetes and hypertension; however, access and use of these were found to be very low among the intended patients.

4. Challenges of record maintenance

In the busy healthcare establishments (all three *taluka* hospitals and some of the busy PHCs), maintenance of the NCD patient records and NCD follow-up patient registers were challenging. In most PHCs, pharmacists and laboratory technicians maintained these records. In less than 40% of intervention PHCs (n=26), at least 75% of patient record books had been issued to patients. Despite

challenges in busy PHCs, 96% of PHCs maintained the NCD register. In only six PHCs, this register had not been maintained for three consecutive months.

5. NCD agenda in ARS meetings

Despite widespread policy-level focus on NCDs, ARS committees rarely discussed NCDs, nor did they fulfil stock-outs (if any) of NCD medicines from budgets available to them. In only nine PHCs (of the 13 intervention PHCs), there was at least one discussion on NCD care at the PHC during an ARS meeting in the intervention period.

6. Human resource shortages at PHCs

This was one of the key issues observed in the study PHCs. In many PHCs, either the doctor or the other staff (laboratory technician, pharmacist, nurse-midwife) were either part-time (due to overseeing multiple facilities at once due to shortages elsewhere) or unavailable. Many patients missed follow-up visits in PHCs where doctors were not available regularly. At one of the study *talukas,* almost half of all the PHCs were running without a doctor. There was no doctor in six PHCs and no pharmacist in seven PHCs out of all 39 study PHCs during our last follow-up visit. Of the 39 study PHCs, only 14 PHCs were doctors available for more than 10 consecutive months, and in 23 PHCs, the pharmacist was available for more than 10 consecutive months.

7. Gaps in essential diagnostics to provide NCD care

Among the 13 arm-A PHCs, 12 had a functional pharmacy, and eight had a functional laboratory. Out of these eight laboratories, only six could test blood glucose. In the 13 Arm-B PHCs, 10 had functional pharmacies, and all had functional laboratories. However, two of the PHCs did not have the ability to test blood glucose levels. In the 13 control PHCs, all had functional pharmacies, nine had functional laboratories, of which three did not have the facility to test blood glucose.
